# Supplementary material for: Multiscale networks in multiple sclerosis
Source: PLoS Comput Biol. 2024 Feb 8;20(2):e1010980. doi: 10.1371/journal.pcbi.1010980 (PMC10852301; doi:10.1371/journal.pcbi.1010980)
Supplement: S1 Methods — (DOCX) [file pcbi.1010980.s001.docx]

**S1 Methods**

**1. Supplementary Methods**

***Brain Magnetic Resonance Imaging***

All images were acquired from 4 centers with distinct 3-tesla systems after standardizing the acquisition protocols and validating dummy scans by the MRI reading center in Berlin. From Center 1 (Barcelona), a three-dimensional (3D) magnetization prepared rapid gradient echo (MPRAGE) sequence, including the upper cervical cord (0.86 x 0.86 x 0.86 mm resolution, repetition time (TR)=1970 ms, echo time (TE)=2.41 ms), an axial T1-weighted post-gadolinium contrast agent sequence (0.31 x 0.31 x 3 mm resolution, TR=390 ms, TE=2.65 ms), and a 3D fluid-attenuated inversion recovery (FLAIR) sequence, including the upper cervical cord (1 x 1 x 1 mm resolution, TR=5000 ms, TE=393 ms) were acquired longitudinally (2 visits) from 60 MS patients using a Tim Trio MRI (Siemens Medical Systems, Erlangen, Germany). From Center 2 (Oslo), a 3D sagittal brain volume (BRAVO) sequence for pre- and post-gadolinium contrast agent administration, including the upper cervical cord (1 x 1 x 1 mm resolution, TR=8.16 ms, TE=3.18 ms), and a 3D FLAIR sequence, including the upper cervical cord (1 x 1 x 1.2 mm resolution, TR=8000 ms, TE=127.254 ms) were acquired longitudinally (2 visits) from 97 MS patients using a Discovery MR750 MRI (GE Medical Systems,). From Center 3 (Berlin), a 3D sagittal MPRAGE sequence, including the upper cervical cord (1 x 1 x 1 mm resolution, TR=1900 ms, TE=3.03 ms), and a 3D FLAIR sequence, including the upper cervical cord (1 x 1 x 1 mm resolution, TR=6000 ms, TE=388 ms) were acquired longitudinally (2 visits) from 87 MS patients using a Tim Trio MRI (Siemens Medical Systems, Erlangen, Germany). From Center 4 (Genova), a sagittal fast-spoiled gradient-echo (FSPGR) sequence, including the upper cervical cord (1 x 1 x 1 mm resolution, TR=7.312 ms, TE=2.996 ms), a 3D turbo field echo (TFE) sequence for post-gadolinium contrast agent administration (1 x 1 x 1 mm resolution, TR=8.67 ms, TE=3.997 ms), and a 3D FLAIR sequence, including the upper cervical cord (1 x 1 x 1 mm resolution, TR=6000 ms, TE=122.162 ms) were acquired longitudinally (2 visits) from 88 MS patients using a Signa HDxt MRI (GE Medical Systems) and Ingenia MRI (Philips Medical Systems).

***MRI Post-processing***

Analysis for all scans were conducted at the MRI reading center in Berlin. Preprocessing included registration to MNI-152 standard space (fslreorient2std), white and gray matter brain masking (Computational Anatomy Toolbox 12 Toolbox for MATLAB SPM12, <http://www.neuro.uni-jena.de/cat/>), N4-bias field correction (Advanced Normalization Tools, <http://stnava.github.io/ANTs/>) and linear, rigid body registration of T2-weighted (FLAIR) images to T1-weighted (MPRAGE, BRAVO, and FSPGR) images (FSL FLIRT, <https://fsl.fmrib.ox.ac.uk/fsl/fslwiki/FLIRT/UserGuide>). Each second session for each patient T1-weighted image and FLAIR image was co-registered to the individual first session using the transformation matrices saved from the first session transformation from native space images to MNI-152 standard space using FSL FLIRT. Post-contrast agent T1-weighted images were also co-registered to MNI-152 standard space and longitudinally when available.

***Brain Lesion Segmentation***

T2-hyperintense lesion segmentation was performed manually on co-registered T1-weighted images and T2-weighted FLAIR images by two experienced MRI technicians from the Berlin center. Lesions were segmented and saved as binary masks using ITK-SNAP (www.itksnap.org). First session lesion masks were subsequently overlayed onto second session co-registered T1-weighted and FLAIR images for editing, to include any T2-hyperintense lesion changes (i.e., new lesions, enlarging lesions, or decreasing lesions) in the follow-up scans. Any discrepancies in co-registrations that were visible between sessions were corrected manually using the ITK-SNAP automated registration tool prior to follow-up lesion mask edits. Binary gadolinium enhancing lesion masks were created manually using the same tools on the postgadolinium T1-weighted MR images by the same two technicians. Lesion counts and volumes were extracted from lesion masks using FSL maths (<https://fsl.fmrib.ox.ac.uk/fsl/fslwiki/Cluster>).

***MRI Analysis***

T2-hyperintense lesion masks were used to fill longitudinally co-registered T1-weighted (not post-gadolinium scans) images using FSL lesion filling (https://fsl.fmrib.ox.ac.uk/fsl/fslwiki/lesion_filling) with white matter masks created from the Computational Anatomy Toolbox for SPM12 (CAT12, <http://www.neuro.uni-jena.de/cat/>). Lesionfilled T1-weighted images were then used for whole brain white and gray matter volume extraction, including the follow-up session percent brain volume change (PBVC) using FSL SIENAX/SIENA (https://fsl.fmrib.ox.ac.uk/fsl/fslwiki/SIENA). The same T1-weighted lesion-filled images were used for whole thalamus volume (sum of left and right thalamic volumes) calculation using FSL FIRST (https://fsl.fmrib.ox.ac.uk/fsl/fslwiki/FIRST). All volumes are reported in milliliters.

***Optical Coherence Tomography***

Retinal OCT scans were performed using the Spectralis device in three centers and the Nidek device at Oslo center. OCTs were collected in eye-tracking mode by trained technicians under standard ambient light conditions (lighting level of 80–100 foot-candles) and without pupillary dilatation. Correction for spherical refractive errors was adjusted prior to each measurement, and the technicians performing OCT scans were aware of the patient’s clinical history. The peripapillary Retinal Nerve Fiber Layer thickness (pRNFL, μm) was measured with a 12-degree diameter ring scan automatically centered on the optic nerve head (100 ART, 1,536 A-scans per B scan). The macular scan protocol involved a 20 x 20-degree horizontal raster scan centered on the fovea, including 25 B scans (ART ≥9, 512 A-scans per B scan). A single grader at the reading center in Berlin performed intra-retinal layer segmentation using Orion software (Voxeleron Inc, Berkeley, US) to quantify the macular ganglion cell plus inner plexiform layer (GCIPL) and the macular inner nuclear layer thicknesses (μm) in the 6 mm ring area as previously described[1]. All OCT scans fulfilled OSCAR-IB criteria[2] and scans with an insufficient signal to noise ratio, or when the retinal thickness algorithm failed were repeated, or the data was ultimately excluded.

***Data Processing***

The omics and clinical datasets were ultimately used to build the multilayer network, where each dataset represents a layer in the network. The data were examined to handle missing values, identify which patients have data from which layers, as well as divided into groups based on gender, disease severity, medication, etc. No imputation was used in this study. Patients were divided into mild and severe groups according to the tertiles of their age-related multiple sclerosis severity (ARMSS) score. Patients in the lower 40th percentile were classified as mild, and those in the upper 40th percentile classified as severe. The 2-year follow-up data from the clinical and imaging layers were used to calculate the change from baseline, and these changes were added as new variables.

**References**

1. Oertel FC, Havla J, Roca-Fernandez A, Lizak N, Zimmermann H, Motamedi S, et al. Retinal ganglion cell loss in neuromyelitis optica: a longitudinal study. J Neurol Neurosurg Psychiatry. 2018;89(12):1259-65. Epub 2018/06/21. doi: 10.1136/jnnp-2018-318382. PubMed PMID: 29921610.

2. Tewarie P, Balk L, Costello F, Green A, Martin R, Schippling S, et al. The OSCAR-IB consensus criteria for retinal OCT quality assessment. PloS ONE. 2012;7(4):e34823. Epub 2012/04/27. doi: 10.1371/journal.pone.0034823

PONE-D-11-21092 [pii]. PubMed PMID: 22536333; PubMed Central PMCID: PMC3334941.

**Figure Legends**

**Figure S1. Pipeline for network build-up for the topological and dynamic networks.**

**Figure S2: Comparing the network topology of various subsets of patients from the original datasets**: (a,b) disease severity, (c,d) disease subtype, (e,f) efficacy of treatment.

**Figure S3: Comparison of paths identified for the various subsets of patients from the original datasets**. The first page of images shows differences between mild and severe patients, the second page relapse-remitting and progressive MS patients, and the third page patients taking low efficacy and high efficacy treatments. In each comparison, sources from three different layers are shown: (a,b) sources from proteomics, (c,d) sources from genomics, and (e,f) sources from cytomics.

**Figure S4. Cytometry plots for the expression of phosphoGSK3AB, phosphoHSBP1 and phosphor RS6 in immune cell subpopulations.** The gating strategy for phospho-flow cytometry analysis. Examples of phospho-GSK3AB, phospho-HSBP1, and phospho-RS6 staining in the immune cell subpopulations for MS patients are presented.

**Figure S5. Overlap between the paths identified in the single-cell analysis and the paths identified in the UNIPROT database.**
