# Supplementary material for: Multiscale networks in multiple sclerosis
Source: PLoS Comput Biol. 2024 Feb 8;20(2):e1010980. doi: 10.1371/journal.pcbi.1010980 (PMC10852301; doi:10.1371/journal.pcbi.1010980)
Supplement: S2 Fig — (PDF) [file pcbi.1010980.s003.pdf]

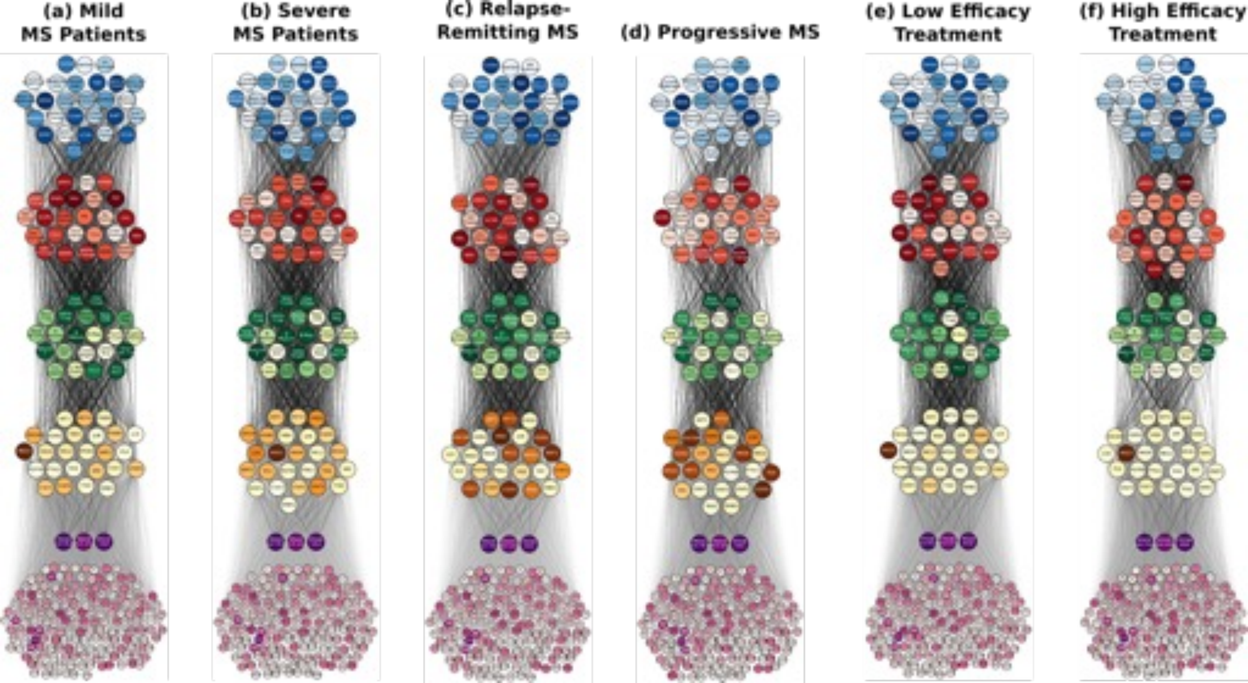
(a) Mild MS Patients
(b) Severe MS Patients
(c) Relapse-Remitting MS
(d) Progressive MS
(e) Low Efficacy Treatment
(f) High Efficacy Treatment
