# Supplementary material for: Multiscale networks in multiple sclerosis
Source: PLoS Comput Biol. 2024 Feb 8;20(2):e1010980. doi: 10.1371/journal.pcbi.1010980 (PMC10852301; doi:10.1371/journal.pcbi.1010980)

GSK

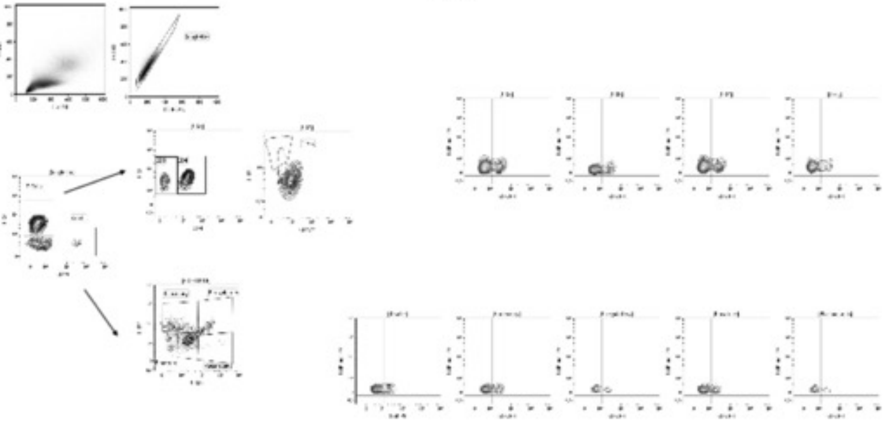

HSBP1

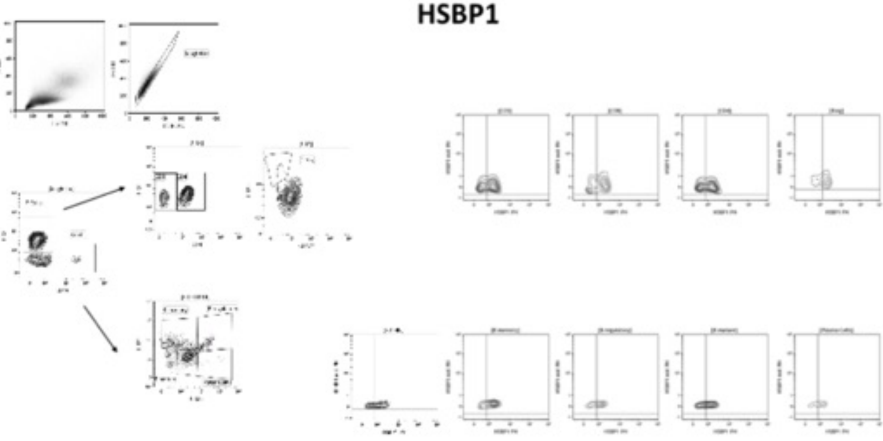

RS6

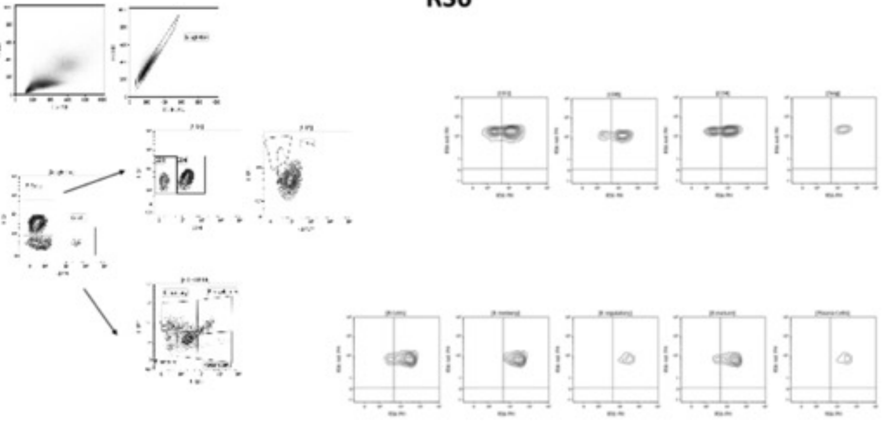

Supplement: S4 Fig — (PDF) [file pcbi.1010980.s005.pdf]
