# Supplementary material for: Multiscale networks in multiple sclerosis
Source: PLoS Comput Biol. 2024 Feb 8;20(2):e1010980. doi: 10.1371/journal.pcbi.1010980 (PMC10852301; doi:10.1371/journal.pcbi.1010980)
Supplement: S5 Fig — (PDF) [file pcbi.1010980.s006.pdf]

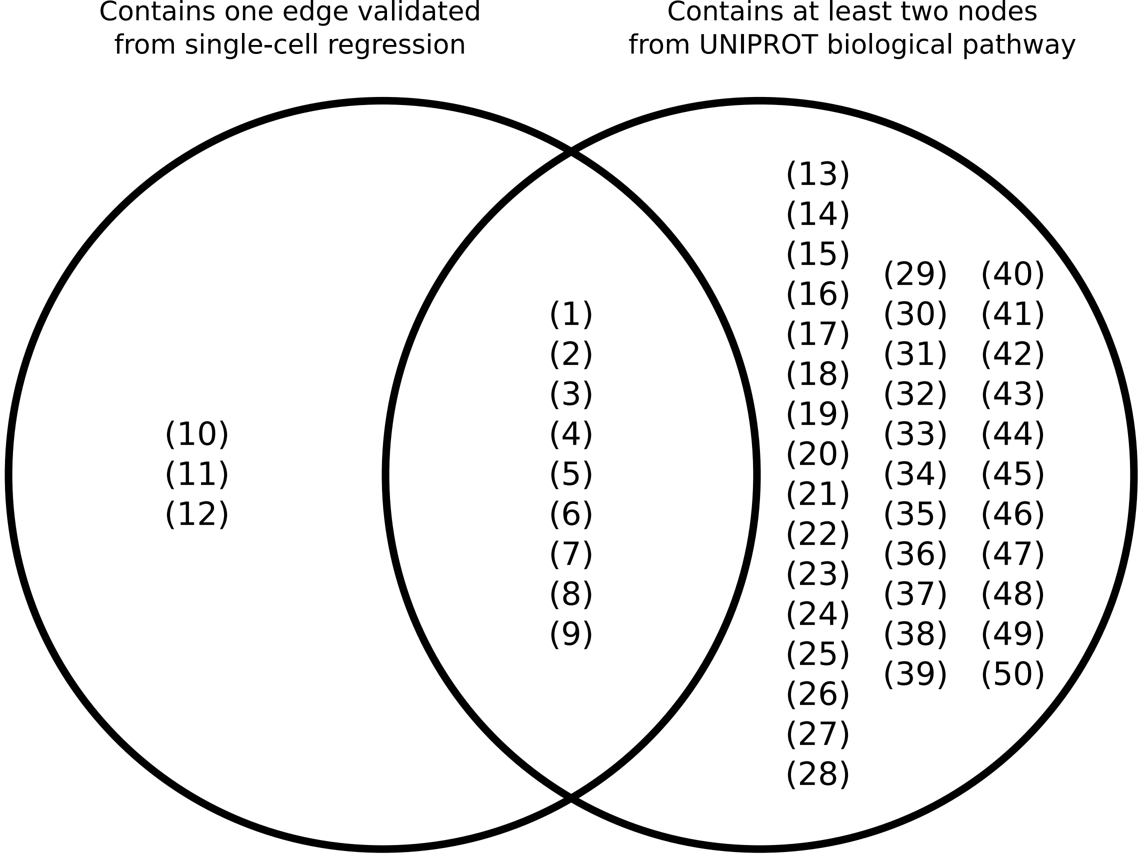

Node within UNIPROT biological pathway
Edge validated by single-cell regression

(A) MK03 > Total T Cells > mRNFL > T25WT
(1) **GSK3AB** - **22:22205353** - 8:128814091 - **MK03** - **Total T Cells - mRNFL** - T25WT
(2) **MP2K1** - 1:11856378 - **MK03** - **Total T Cells - mRNFL** - T25WT

(B) HSPB1 > B Memory > NBV > T25WT
(3) **CREB1** - **22:40291807** - HSPB1 - B Memory - **NBV - T25WT**

(C) STAT6 > Th17 > NGMV Change > Years with Disease
(4) MSGB non-HLA - **10:94479107** - 10:31395761 - **STAT6** - Th17 - **NGMV Change - Years with Disease**
(5) PGFRB - 3:121783015 - **P53** - **STAT6** - Th17 - **NGMV Change - Years with Disease**
(6) **PGFRB** - 3:121783015 - **P53** - STAT6 - Th17 - **NGMV Change - Years with Disease**

(D) KS6B1 - LCK > Total T Cells - Th1 Non Classic > NGMV - T2LV > LCVA Change - MSSS - Years since Relapse
(7) **CREB1** - 7:138729795 - 11:122518525 - **KS6B1** - **Total T Cells - NGMV** - Years since Relapse
(8) **STAT1** - 11:95421830 - 11:122518525 - **KS6B1** - **Total T Cells - NGMV** - LCVA Change
(9) **STAT1** - 11:95421830 - 11:122518525 - **KS6B1** - **Total T Cells - NGMV** - Years since Relapse
(10) B Memory - Atypical B Memory - KS6B1 - **Total T Cells - NGMV** - Years since Relapse
(11) MSGB non-HLA - 10:94479107 - 11:118743286 - KS6B1 - **Total T Cells - NGMV** - LCVA Change
(12) MSGB non-HLA - 10:94479107 - 11:118743286 - KS6B1 - **Total T Cells - NGMV** - Years since Relapse

(E) MP2K1 - STAT6 > Th17 > INL > EDSS Change
(13) **AKT1** - 11:47360412 - **SRC** - STAT6 - Th17 - INL - EDSS Change - EDSS Increase?
(14) **CREB1** - 22:40291807 - **MP2K1** - Th17 - INL - EDSS Change
(15) **GSK3AB** - **22:22205353** - 22:37258986 - **MP2K1** - Th17 - INL - EDSS Change
(16) **MK03** - 7:128573967 - 22:37258986 - **MP2K1** - Th17 - INL - EDSS Change
(17) **MK03** - 16:79350204 - 1:11856378 - **MP2K1** - Th17 - INL - EDSS Change
(18) **MK03** - 1:11856378 - **MP2K1** - Th17 - INL - EDSS Change - EDSS Increase?
(19) MSGB non-HLA - **10:94479107** - 3:105455955 - **STAT6** - Th17 - INL - EDSS Change
(20) MSGB non-HLA - **10:94479107** - 10:31395761 - **STAT6** - Th17 - INL - EDSS Change
(21) MSGB non-HLA - 10:94479107 - **11:118743286** - **MP2K1** - Th17 - INL - EDSS Change
(22) **NRF2** - 17:57859210 - **STAT6** - Th17 - INL - EDSS Change - EDSS Increase?
(23) **P53** - 2:68646536 - **19:18301979** - **MP2K1** - Th17 - INL - EDSS Change
(24) **PTN11** - 19:11173928 - 22:37258986 - **MP2K1** - Th17 - INL - EDSS Change
(25) **PTN11** - 19:11173928 - 5:141539339 - **MP2K1** - Th17 - INL - EDSS Change
(26) **SRC** - **10:75653800** - 1:157686337 - MP2K1 - Th17 - INL - EDSS Change
(27) STAT3 - **STAT1** - **STAT6** - Th17 - INL - EDSS Change
(28) **WNK1** - 4:103911781 - **3:105455955** - STAT6 - Th17 - INL - EDSS Change

(F) MP2K1 > CD8 Treg > GCIPL > EDSS Change
(29) **CREB1** - 22:40291807 - **MP2K1** - CD8 Treg - GCIPL - EDSS Change
(30) GSK3AB - **22:22205353** - 22:37258986 - **MP2K1** - CD8 Treg - GCIPL - EDSS Change
(31) **GSK3AB** - **22:22205353** - 22:37258986 - MP2K1 - CD8 Treg - GCIPL - EDSS Change
(32) **MK03** - 7:128573967 - 22:37258986 - **MP2K1** - CD8 Treg - GCIPL - EDSS Change
(33) **MK03** - 1:11856378 - **MP2K1** - CD8 Treg - GCIPL - EDSS Change - EDSS Increase?
(34) **P53** - 2:68646536 - 19:18301979 - **MP2K1** - CD8 Treg - GCIPL - EDSS Change
(35) **P53** - 2:68646536 - **19:18301979** - MP2K1 - CD8 Treg - GCIPL - EDSS Change
(36) **PTN11** - 19:11173928 - 22:37258986 - **MP2K1** - CD8 Treg - GCIPL - EDSS Change
(37) **SRC** - 10:75653800 - 1:157686337 - **MP2K1** - CD8 Treg - GCIPL - EDSS Change
(38) **SRC** - **10:75653800** - 1:157686337 - MP2K1 - CD8 Treg - GCIPL - EDSS Change
(39) STAT3 - **STAT1** - **MP2K1** - CD8 Treg - GCIPL - EDSS Change

(G) MP2K1 - STAT6 > Th17 > mRNFL > T25WT - ARMSS
(40) **CREB1** - **22:40291807** - **MP2K1** - Th17 - mRNFL - T25WT - Years since Relapse
(41) **MK03** - **7:128573967** - 22:37258986 - MP2K1 - Th17 - mRNFL - T25WT
(42) **MK03** - 7:128573967 - 22:37258986 - **MP2K1** - Th17 - mRNFL - T25WT
(43) MSGB non-HLA - 10:94479107 - **11:118743286** - **MP2K1** - Th17 - mRNFL - T25WT
(44) MSGB non-HLA - **10:94479107** - **10:31395761** - **STAT6** - Th17 - mRNFL - T25WT
(45) **NRF2** - 17:57859210 - **MP2K1** - Th17 - mRNFL – T25WT
(46) **PTN11** - 19:11173928 - 22:37258986 - **MP2K1** - Th17 - mRNFL - T25WT
(47) **SRC** - **10:75653800** - 1:157686337 - MP2K1 - Th17 - mRNFL - T25WT
(48) **WNK1** - 4:103911781 - **SRC** - STAT6 - Th17 - mRNFL - T25WT
(49) **WNK1** - 4:103911781 - **3:105455955** - STAT6 - Th17 - mRNFL - T25WT

(H) Atypical B Memory - B Memory - Th1 Classic > mRNFL - T2LV > EDSS - T25WT
(50) MSGB non-HLA - **10:94479107** - **10:31395761** - HSPB1 - B Memory
