# Supplementary material for: Multiscale networks in multiple sclerosis
Source: PLoS Comput Biol. 2024 Feb 8;20(2):e1010980. doi: 10.1371/journal.pcbi.1010980 (PMC10852301; doi:10.1371/journal.pcbi.1010980)
Supplement: Attachment — Submitted filename: response to reviewers PloS Comp Biol R1.docx [file pcbi.1010980.s016.docx]

**Response to the editor**

Dear Prof Zhaolei Zhang

Attached we are submitting the revised version of our manuscript following your indications and after having addressed the reviewer comments. We have focused on the main question raised from reviewers which is the validation of our algorithm. Because there are not validated studies of this kind in the same disease (MS), we made use of a previously published study from Prof Michael Snyder from Stanford in patients with Diabetes suffering an infection. We have applied our approach to this dataset and show we are able to identify the same pathways than described in the original study as well as providing new insights about complex interactions between biological scales. Such results are a strong endorsement that our algorithm is able to identify the flow of biological information across scales relevant for the biological process under study (MS or infections in diabetes). Second, we have paid attention to the quality and accessibility of the figures, upgrading the previous ones and providing new ones that will help to visualize the results of our study and the biological paths identified. We hope such new results and visualizations are appreciated by the reviewers as satisfactory. We would also be delighted to follow your advice for improving the readability and message of our article to improve the reader experience.

Below we provide a point-by-point answer to the reviewer comments. Page numbers refers to the tracked changes version.

**Reviewer #1**

This paper presents a network analysis using multiomics data to discover biological pathways associated with multiple sclerosis (MS), ranging from SNPs to clinical phenotypes. The authors constructed multilayer networks that interconnected feature networks of individual omics from a designated cohort, including SNPs, genes & proteins, cell types, endophenotypes from MRIs and OCTs, as well as clinical phenotypes. They prioritized cross-layer pathways using Boolean simulation to identify critical paths, such as linking specific proteins to immune cell types to retinal nerve fiber layer thickness to MS severity. Some of these pathways were validated using single-cell data.

In general, the study presents an interesting application of network biology to understand MS biology. However, several details, particularly in the methods section, are insufficiently described, making it difficult to evaluate the rigor of this study. The rationale for network selection and construction is also unclear. Additionally, the manuscript requires further editing to improve clarity. Here, I just highlight some major concerns.

1. Multiomics data. It remains unclear whether the multiomics data were obtained from the same individuals in the cohort. It appears that certain omics layers heavily relied on public databases, which may not be specific to MS.

All the data used in the MS study has been obtained from the same cohort of MS patients. The only data obtained from databases was the gene regulatory network. Such database info was used to provide the network context for the SNPs and be able to conduct a similar analysis for genetic networks than the other networks. This was required because the dichotomic nature of the SNPs than prevented having a continuous variable enabling to calculate the mutual information between nodes. The approach of using a network topology from a reference database was used before in our study “Prediction of combination therapies based on topological modeling of the immune signaling network in multiple sclerosis” (M. Bernardo-Faura et al. Genome Med. 2021 Jul 16;13(1):117). We have added the following text to the main manuscript (page 15): “All data used in this study is coming from the study’s cohort except for the reference Genomic Regulatory Network used to embed the SNP data.”

2. Network construction. How to interconnect individual networks using mutual information again is unclear. It is unclear how the Boolean simulation would be sensitive to mutual information cutoffs. Moreover, it is unclear why Figure 3 switched back to correlation instead of mutual information, considering that proteins and genes likely exhibit strong nonlinear relationships. In sum, the authors did not provide sufficient details about their methodology.

We have explained better the analyses in which we used mutual information and which we use Pearson correlation. Mutual information was used for the first part of the study, the structural / topological analysis, allowing standard network analysis (degree, density). Mutual information was chosen because it allowed to capture linear and non-linear dependencies and for this reason the topological analysis will be more comprehensive. For the dynamic analysis (Boolean analysis aimed to identified paths), we used Pearson correlation because it provides positive and negative values for the edges weight, which was required for the Boolean simulations as AND or OR gates (at the price of losing the non-linear correlations). Now we have created the figure shown below and added a text explaining this pipeline better.

*Figure added to manuscript within the graphical abstract:*

*We added such explanation to the manuscript (page 15 and 19), supplementary material (page 13) and the figure to the supplementary methods:*

***Multilayer networks in MS (page 15)***

At this stage, mutual information is used due to its ability to detect nonlinear correlations between two elements.

We sought to connect the elements from the various layers together. Edges were again defined using mutual information, but this time two elements from any of the five layers were connected. At this step, hierarchy among the layers is ignored to gain a sense of how the network is connected regardless of scale.

***Dynamic network analysis identifies gene-protein-cell paths associated with phenotype (page 19)***

Pearson correlation was used for the dynamic network analysis instead of mutual information to identify the stimulatory or inhibitory nature of the interaction between elements, which is required for the Boolean simulation

3. Gene regulatory network. It remains unclear how specific the public gene regulatory networks used in the study are to MS, particularly linking SNPs to genes. The underlying gene regulatory mechanisms for constructing edges would be more useful than mutual information, such as SNPs interrupting transcription factor bindings and chromatin interactions to regulate downstream target genes.

The use of the gene regulatory network (GRN) is intended to provide a network where to integrate the binary SNPs data from MS patients. This was not required for the other types of data, because they were quantitative, and the networks can be created directly from the data. However, this was not the case for SNPs, because they are binary. Then, we provide a default genetic network were to project our SNP data. It is true that the GRN comes from healthy control samples, but its intended use is to provide a reference network to integrate the SNPs from the patients and the experimental data will drive the weights of the edges and the degree of the nodes. This approach of using a network topology form a database was used before in our study “Prediction of combination therapies based on topological modeling of the immune signaling network in multiple sclerosis” (M. Bernardo-Faura et al. Genome Med. 2021 Jul 16;13(1):117). Since the SNP information is binary, the relationships from the GRN adds a topology that is used for the dynamic behavior.

*Text added to manuscript (page 52):*

*Expanded genetic network including regulatory network information*

The underlying GRN provides a reference network of how the genes interact in the healthy state. The allelic (SNP) information alone is binary and does not change throughout time, limiting the analysis. Considering the underlying GRN strengthens the analysis by allowing us to integrate the MS-related SNPs into a model that considers dynamic behavior. Similar approaches have been seen in previous studies, where a previous knowledge network of protein signaling interactions is used as a base for logic modeling that incorporates new data to determine the differences between healthy and diseased states (M. Bernardo-Faura *et al.* 2021).

4. Biology. While MS is known to be related to immunology, other cell types, such as neurons, also play important roles. Given the network biology approach, neglecting such crucial cell types would be a significant miss.

We agree with the reviewer that CNS cells are key for modeling how MS damage the CNS. However, collecting CNS cells and conducting omics with them is extremely difficult in patients. By contrast, PBMCs will reflect the status of the immune response driving the inflammatory attacks. Considering that MS is an autoimmune disease, by studying multiple omics from blood, we would be able to capture signatures of the autoimmune process. Regarding the role of the CNS damage (neurons and glia) and considering the difficulties to access such samples, we rely on imaging for obtaining relevant information about the target tissue. Then, our compromise was interrogating the immune system with omics and the CNS status with imaging. We have acknowledged such limitations in the discussion.

Text added to manuscript

Methods: *Sys4MS cohort (page 42)*:

The imaging data is aimed to capture the damage of the CNS, and the immune cells data (genomics, proteomics, and cytomics) aims to capture the dynamics of the immune system during the autoimmune response (Cellerino, M. et al. 2020) (James, T. et al. 2018) (Bernardo-Faura, M. et al. 2021).

Discussion (page 42)

We were unable to collect cells from the CNS for omics analysis and rely on imaging data for modeling CNS damage, which is an approximation to the problem.

5. Writing. The manuscript would benefit from harmonizing the writing style across different sections to improve overall clarity.

We have reviewed the overall manuscript to ensure the writing style is now more homogenous.

**Reviewer #2**

Overall:
This work is timely and speaks to an important goal in understanding disease mechanism: how to integrate across multiple layers of data and identify correlations across layers to make a causal inference across these. The work has promising conceptual strengths, which when executed rigorously, could help advance the field of systems biology of disease. However, in the current form, the work provides insufficient information to help the reader evaluate the validity of the methods, and significance of the results. I recommend that this paper be re-evaluated after major revisions to help the community better evaluate the significance of this work.
Strengths: They use a multi-modal dataset in MS that is large by contemporary scales, considering all the layers profiled. They have data from five layers profiled, including clinical, genomic and imaging layers. The work is innovative in proposing the use of mutual information and Boolean gating to create a multi-layered network and infer cross-layer correlation. This is an important methodological idea that needs to be developed (see recommendations for revision).
They attempt to validate the cross-layer edges identified by their Boolean analysis by linear regression between phosphoproteomic values and phenotypes scores.
Major revisions:
1. My biggest concern with this work is that the algorithm used to infer pathways seems to be completely new, and no benchmarking is shown to convince the reader that:

We agree with the reviewer that this is a fundamental aspect for our approach. The algorithm in this study was based on that from our previous study “Relaxation dynamics and frequency response of a noisy cell signaling network” (Domedel et al. (30). Because there are no other similar datasets from MS patients, nor validated pathways identified with this approach, we have conducted a validation of the algorithm using an independent dataset published by Prof Michael Snyder from Stanford University (Zhou et al. Nature. 2019 May;569(7758):663-671). Using such a dataset and by applying our algorithm for multi-layer network analysis, we validated many of the paths identified in this study and provide new paths highlighting the ability of our approach for identifying paths across biological scales (see below for a fool description of the dataset and our analysis).

*Section added to manuscript (page 36):*

***Algorithm validation in an independent diabetes and infectious disease dataset***

Validation of the path identification algorithm was performed using datasets from the study *Longitudinal multi-omics of host-microbe dynamics in prediabetes* (Zhou et al. 2019). These datasets included six layers total: RNA-seq, cytokines, general proteins, nasal microbes, gut microbes, and clinical tests. The original data is longitudinal, where data was collected periodically from patients over the course of several years. These included healthy visits and visits where the patient had an upper respiratory infection. Samples of these visits were taken so each group (healthy and infected) contained a visit from each patient. Not all patients had a visit with an infection, so the data is skewed to healthy (n=84) and infected (n=25). Networks were created from each of the six datasets. Top paths were identified through Boolean simulations in the same manner as the Sys4MS datasets.

The paths identified from the Boolean simulations were compared with molecules from canonical pathways that were curated in the diabetes study (Zhou et al. 2019). These pathways include acute phase response signaling, neuroinflammation signaling, and Th1 and Th2 activation. Molecules from these pathways are present in the RNA-seq, cytokine, and protein datasets. The Boolean paths identified in this study containing at least two molecules from different layers from a given canonical pathway were considered.

By applying our algorithm to the diabetes dataset we found significant Pearson correlations between nodes of the Boolean paths with respect to the various canonical pathways. In many cases, the Pearson correlation was higher in the paths identified in the infected case compared to the healthy case. These Boolean paths contained molecules present in the following canonical pathways: 1) *Acute phase response signaling;* 2) *Huntington's disease signaling*; 3) *Neuroinflammation signaling*; 4) *Role of JAK1 and JAK3 in gamma-c cytokine signaling*; 5) *Role of macrophages, fibroblast, and endothelial cells in rheumatoid arthritis*; 6) *Role of pattern recognition receptors in recognition of bacteria and viruses*; 7) *Role of tissue factor in cancer*; and 8) *T helper cell differentiation*. Visualizations of the Boolean paths that contain molecules from sample canonical pathways are available in Figure 9.

Further paths can be seen at [https://keithtopher.github.io/canonial_pathways/](https://keithtopher.github.io/fivelayer_pathways/).

**Supplementary Figure S5: Difference in Pearson correlation between healthy and infected cases.** The networks shown contain paths that were identified from the Boolean simulations in the infected network. Furthermore, each path contains at least two nodes from two different layers that are present in the acute phase response signaling biological pathway. The same paths do not necessarily appear in the healthy network, so edges with Pearson correlation are shown. There is a notable increase in the strength of the connections, both positive and negative, in the infected case.

a. the method has a reasonable sensitivity/specificity and will indeed highlight true associations while not identifying false associations
b. The method works equally well on the diverse data types shown - SNPs, imaging data and clinical data have different data characteristics. How do we know it is valid to apply this strategy to all these diverse datatypes and get something meaningful?

We agree that data type and the underlying biology is quite different between modalities. This is the reason why we are using mutual information for the topological analysis, to capture information distribution for linear and nonlinear systems. Indeed, the fact that the density analysis confirmed that nodes are preferentially attached to their modality suggests that mutual information and Boolean modeling is able to adequately manage the diversity of the data type. Moreover, some datatypes such as SNPs were very difficult to include in the network analysis because of their binary nature. For this reason we make use of additional strategies such as providing a reference quantitative network such as regulatory networks and using the polygenic risk scores (MSGB) for the topological analysis. We also normalized the phosphoproteomics data to avoid skewed data. Finally, by applying this algorithm to an independent dataset from infectious diseases and retrieving similar results than the original study plus additional pathways, suggest the analysis strategy is robust enough across diverse biological datatypes. (Page 8, 17, 51, 52)

c. The method accurately groups patients with similar clinical profiles together. E.g., Do patients with the same type of MS co-cluster together?

We have conducted an analysis comparing different disease subtypes such as progressive vs relapsing MS, mild to severe disability groups, or low efficacy vs high efficacy treated patients observing that within each subgroup the profiles were more similar than between categories. These results indicates that the MS subtypes cluster together. In the supplementary information we show the comparison between disease subtype, disease severity and use of therapies groups for both the topological network analysis.

*Figures added to supplementary (page 22):*

**Supplementary Figure S2: The multi-layer networks by subgroups:** (a,b) disease severity, (c,d) disease subtype, (e,f) efficacy of treatment.

***Identified paths for networks of disease severity, disease subtype, and efficacy of treatment***

**Supplementary Figure S3: Comparison of paths identified for the various subsets of patients from the original datasets.** The images shows differences between mild and severe patients, relapse-remitting and progressive MS patients, and low efficacy and high efficacy treatments. In each comparison, sources from three different layers are shown: (a,b) sources from proteomics, (c,d) sources from genomics, and (e,f) sources from cytomics.

d. This comment applies to the mutual information networks and the Boolean flow inference work. This method is the foundation of all inferences in this paper. The authors need to show a proof-of-concept of some kind, e.g., apply this new algorithm to an existing multi-modal dataset (e.g., TCGA breast cancer data) and show that the method captures known pathways (SNP > gene > protein > pathway > phenotype, at the least). Without some kind of reference point, it is hard to believe the validity of this method and I would not recommend the publication of this work.

As explained above and following the reviewer indications we have applied our algorithm for path identification to a dataset from the study *Longitudinal multi-omics of host–microbe dynamics in prediabetes* (Zhou et al. Nature. 2019 May;569(7758):663-671). The raw data was provided by Prof Snyder from Stanford University and included multilayer data from healthy subjects and from infected patient data. The paths identified with our analysis were compared to the canonical biological pathways (Gene Ontology) they provided as the main results. Results for this validation are described in the manuscript as a summary and in the supplementary material as extended version (see above). We used the Gene Ontology as reference to validate paths identified in this study to be identified by our study (supplementary tables 10 and 11 from the original Snyder’s article).

*Section added to manuscript (page 29, 36):*

***Algorithm validation in an independent diabetes and infectious disease dataset***

Validation of the path identification algorithm was performed using datasets from the study *Longitudinal multi-omics of host-microbe dynamics in prediabetes* (Zhou et al. 2019). These datasets included six layers total: RNA-seq, cytokines, general proteins, nasal microbes, gut microbes, and clinical tests. The original data is longitudinal, where data was collected periodically from patients over the course of several years. These included healthy visits and visits where the patient had an upper respiratory infection. Samples of these visits were taken so each group (healthy and infected) contained a visit from each patient. Not all patients had a visit with an infection, so the data is skewed to healthy (n=84) and infected (n=25). Networks were created from each of the six datasets. Top paths were identified through Boolean simulations in the same manner as the Sys4MS datasets.

The paths identified from the Boolean simulations were compared with molecules from canonical pathways that were curated previously (Zhou et al. 2019). These pathways include acute phase response signaling, neuroinflammation signaling, and Th1 and Th2 activation. Molecules from these pathways are present in the RNA-seq, cytokine, and protein datasets. The Boolean paths identified in this study containing at least two molecules from different layers from a given canonical pathway were considered.

Notable differences were seen in the strength of the Pearson correlation between nodes of the Boolean paths with respect to the various canonical pathways. In many cases, the Pearson correlation was higher in the paths identified in the infected case compared to the healthy case. These Boolean paths contained molecules present in the following canonical pathways: 1) *Acute phase response signaling;* 2) *Huntington's disease signaling*; 3) *Neuroinflammation signaling*; 4) *Role of JAK1 and JAK3 in gamma-c cytokine signaling*; 5) *Role of macrophages, fibroblast, and endothelial cells in rheumatoid arthritis*; 6) *Role of pattern recognition receptors in recognition of bacteria and viruses*; 7) *Role of tissue factor in cancer*; and 8) *T helper cell differentiation*. Visualizations of the Boolean paths that contain molecules from sample canonical pathways are available in **Figure 9**. Further paths can be seen at [https://keithtopher.github.io/canonical_pathways/](https://keithtopher.github.io/fivelayer_pathways/).

**Figure 9: Difference in Pearson correlation between healthy and infected cases.** The networks shown contain paths that were identified from the Boolean simulations in the infected network. Furthermore, each path contains at least two nodes from two different layers that are present in the acute phase response signaling biological pathway. The same paths do not necessarily appear in the healthy network, so edges with Pearson correlation are shown. There is a notable increase in the strength of the connections, both positive and negative, in the infected case.


2. Although the authors attempt to validate pathway links using regression, there is no clear presentation of what the sensitivity and specificity of the validation is. Part of the problem is the presentation. The pathways identified are simply listed in bullet points in the text. The validation is limited to one indirect link (phosphoproteomics to phenotype, but multiple phenotypic variables are compared - multiple testing correction?), and to correlation. In its current state, too much effort is required on the reader’s part to understand how many of the pathways and links identified by their Boolean logic were validated and how to evaluate the accuracy of the method and the results.

Following the reviewer's suggestions, in the new version we provide the results from the linear regressions in figures 5a,b,c to make easier for the reader, indicating which pathways are validated (page 37). The figure is shown below (in the reviewer’s next point) along with connections to biological pathways. The information about all the paths and their connections to biological pathways is provided in Supplementary File S6.

I recommend: Create a table in which you list all the pathways identified (same ones as mentioned in the body of the text). Then: 1) add a column to indicate which of these were validated by the regression analysis and which were not validated (yes/no), and 2) add a column to indicate whether the path is known in the literature (cite). Add a Venn diagram showing the overlap between “known links in literature” and “predicted links” Discuss these factors in the Discussion section. Without this reference point for the reader, the presentation of this result makes it hard to evaluate what the added benefit of the multi-layer networks is.

We truly appreciate the reviewer's suggestions. Following his advice, we have included new figure 5a,b,c and supplementary tables listing all the pathways indicating if they are validated and if they have been published before (with the references). Due to the high number of paths, showing the overlap with canonical pathways for all identified paths was not feasible. Therefore, one is shown as a figure in the main text, and the rest are in Supplementary File S6. Let us clarify that only genes and proteins can be validated with Gene Ontology, but not cells, imaging or phenotypes. For the same reason, we analyzed the connections to biological pathways, but only for the genes and proteins being part of the GO pathways. We acknowledge such limitations in the manuscript.

Section added to manuscript (page 34):

***Comparison of paths with known biological pathways***

The paths identified from the Boolean simulations were compared to known biological pathways obtained from the Uniprot database[32]. Specifically, we searched for paths that contained at least two nodes that were part of the same biological pathway. In figure 8 we show the overlap between the paths identified in our analysis that contain one edge validated in the single-cell analysis and the paths from UNIPROT that contain at least two nodes from UNIPROT biological pathways. We found that four out of 10 paths were present on both datasets, whereas 2 paths were present only in our single-cell analysis and 4 were only observed in the UNIPROT database. The full list of details about paths and their connections to biological pathways are given in File S6 and figure S5. Furthermore, one of the edges that connects the cytomics and imaging layers (Total T Cells - mRNFL) was validated by the single-cell regression analysis

Figure 8. Venn diagram describing the overlap between the paths identified in the single-cell analysis and the paths identified in the UNIPROT database. (1) CD56 Neg > INL - mRNFL > EDSS - T25WT; (2) Total CD8 > NGMV - T2LV > EDSS - 9HPT – SDMT; (3) MK03 > Total T Cells > mRNFL > T25WT; (4) HSPB1 > B Memory > NBV > T25WT; (5) STAT6 > Th17 > NGMV Change > Years with Disease; (6) KS6B1 - LCK > Total T Cells - Th1 Non Classic > NGMV - T2LV> LCVA Change - MSSS - Years since Relapse; (7) MP2K1 - STAT6 > Th17 > mRNFL > T25WT - ARMSS (8) MP2K1 - STAT6 > Th17 > INL > EDSS Change; (9) MP2K1 > CD8 Treg > GCIPL > EDSS Change; (10) Atypical B Memory – B Memory – Th1 Classic > mRNFL – T2LV > EDSS – T25WT.

**Figure 9: Connection of the path from Boolean simulations with biological pathways (Path (1)) and validation by single-cell analysis**. Highlighted in the red and blue boxes are the nodes that are involved with the biological pathways written inside. Highlighted in the green box is the edge that was validated by single-cell regression.

Text added to the manuscript, ***Discussion (Page 38)***:

As shown in **Figure 9**, the link between Total T Cells and mRNFL was validated for Path (1). The data from the single-cell validation concerned the amount of GSK3AB present in the cells, and one of the subpaths of Path (1) indeed contains GSK3AB. This helps in justifying the makeup of the entire path seen in **Figure 9**. Furthermore, nodes from Path (1) are involved in a number of biological pathways, suggesting potential mechanisms for how disruptions in this path can have a negative effect on the organism scale. The validation of Path (1) helps to show how processes at smaller scales (the biological pathways) manifest themselves in the larger scales (cell counts, tissue damage, and walking speed).

3. Figures have detailed colorful networks without any guidance as to the message of each panel is. a) Unclear what the message of Figure 1b-g is. There is no visible substructure. There is no attempt in the text to link network topology to disease features or subtypes. What is the take-home message of these panels?

In the new version we provide guidance to follow the networks’ descriptions (page 9). We would like to clarify that Figure 1 is for visualization purposes of the overall networks, and the detailed examination of the networks is provided in the Github link which allows manipulating and zooming the network. Indeed, we provide an overall summary and message of this section of the network analysis (page 16) as well as providing figures by disease subtype (PMS and RMS) in the supplementary results (page 22). The main message is that the data is more connected within each layer (data type) and that some networks are more highly connected than others based on the nature of the data itself.

Text added to manuscript in Figure 1 caption (page 9):

These networks are meant to show the nodes that are more highly correlated with other nodes in the network. They provide the base to examine the topological structure of the overall multilayer network.

Text added to manuscript, section ***Multilayer networks in MS*** (page 16)

The networks seen in **Figure 1** allow us to see which nodes are more highly connected with the other nodes in the datasets. Nodes with more connections imply they are more highly correlated with their neighbors. Changes in one hub node can signal changes in many other nodes and serve as a means of prediction. For each layer, the hub nodes are as follows:

| Layer | Hub Nodes |
| --- | --- |
| Clinical | age  expanded disability status score (EDSS)  symbol digit modality test (SDMT) |
| Imaging | outer nuclear layer (ORL)  total macular volume (TMV)  macular retinal nerve fiber layer (mRNFL) |
| Cytomics | Naive T-regulatory  Natural T Cells  CD8 T-regulatory |
| Proteomics | STAT6  KS6B1  CREB1 |
| Genomics | MSGB total  MSGB HLA only  MSGB non-HLA |

b) Why are the nodes colored by degree, what is the significance of this? Why aren't they connected by similarity?
The degree is key for defining the small-word structure of the networks. Degree and edge strength are two basic parameters of networks that define their structure and this is the reason why we have used a color code for the degree (with specific color for each layer) and gray scale for the edge weight. Similarity (correlation) is provided as the edge strength.

4. Please revise the abstract to tell us: What is the major conclusion of this paper? What new pathway has been identified in this work that was validated? How has this work improved the mechanistic understanding of MS? Currently the abstract seems to simply say that some results were found, without providing broader context to place the significance of the work.

In the new version, we summarize the main results of the article as the list of pathways identified in MS as relevant for the autoimmune process.

Text added to the manuscript, ***Summary (Abstract)*** (page 4):

The path more commonly found from the Boolean simulations connects protein MK03, with Total T cells, the thickness of the retinal nerve fiber layer (RNFL), and the walking speed. This path contains nodes involved in protein phosphorylation, glial cell differentiation, and regulation of stress-activated MAPK cascade, among others. Specific paths identified were subsequently analyzed by flow cytometry at the single-cell level. Combinations of several proteins (GSK3AB, HSBP1 or RS6) and immune cells (Th17, Th1 non-classic, CD8, CD8 Treg, CD56 neg, and B memory) were part of the paths explaining the clinical phenotype. The advantage of the path identified from the Boolean simulations is that it connects information about these known biological pathways with the layers at higher scales (retina damage and disability). Overall, the identified paths provide a means to connect the molecular aspects of MS with the overall phenotype.

Minor:
1. Too many acronyms, hard to read the work. Please expand acronyms right in Table 1 instead of putting in the footer. First time you use any acronym, please use full form.
We have removed the unnecessary acronyms and spell it in table 1 but kept genes and proteins acronyms because the name is not so informative, and it will make the writing too long. We will follow the editor guidance on this matter to adequate to the journal requirements (page 12, 14).

2. In all the figures, the node labels are too small to read, and it is not possible to draw any inference from these. Please remove or make bigger.

In the figures we need to use acronyms to make them readable. We have improved the size and refer to the Github link for better resolution. In addition, we are providing the images as high resolution pdf that allows to zoom in without losing resolution.

3. The validation is shown for three proteins, but the abstract lists many more. Why is this? It seems inconsistent. Why not just talk about what you validated?

Due to experimental restraints, only a few proteins could be validated with the single-cell analysis. Now, we indicate only the paths that involved the three validated proteins.

4. In the introduction and abstract the authors mention that systems-level function is important for complex diseases. This is not just true about diseases; it is true about any phenotype for a multi-layered organism. Suggest changing this to reflect the fact.

We agree. We have added the sentence “In fact, any phenotype for an organism is dictated by the interplay among these scales.” (page 4)

5. Visualization: The authors use different colors to represent the same variable (confusingly, this is node degree) for different data types. The colour choices also have no relationship to the position of the layer in the system (e.g., lighter is closer to genetic, darker is closer to higher-order phenotypes). While divergent colors are pleasing to the eye they do not contribute to the informative visual exploration of the data.

We have checked that the color is consistent for the datatype in all figures (purple=genes, orange=proteins, green=cells, red=imaging, blue=clinical). The gradient of the color shows the degree of the node, which we felt was useful to show how strongly that node was connected in the network. The gray to dark shading of the edges refers to the links’ strength. It is useful for showing hub nodes, and those that are likely to appear in the later path analysis. In any case, we would follow reviewer and editor indications in how to make it clearer for the reader.

**Reviewer #3**

In this manuscript from Kennedy et al., the authors analyze multiple layers of omics and clinical data from a large prospective cohort of MS patients and healthy controls. They use network analyses to identify salient networks within layers. They then perform interesting and novel analyses to integrate information across layers. This represents an impressive dataset and analytical approach. However, I found the manuscript at times hard to understand and there are some major concerns that need to be addressed.
- As someone who is not an expert in network analyses, I had a lot of difficulty following many of the methods and approaches used. It would be helpful to have a less technical description of the analytical approaches used.

Thank you for your comment. We will be happy to simplify the methods after finishing the revision process based on editor feedback. However, other reviewers have requested expanding the methods and explanations. Then, we will follow the editor indications here.

- I found many of the figures to be hard to follow. There are many figures with a lot of networks and connections between networks, but the figure sizes are so small and it’s hard to know what is important in each figure. Also, I have no idea what figure 3C is trying to show. Rather than showing a lot of network graphs, it would be helpful to have a different approach for visualizing the results so readers can better understand the magnitude of effects. For example, take a SNP identified in the analyses, and show the changes in genomics, proteomics, clinical outcomes as a function of the SNP carrier status.

Our analysis is based on network analysis and for this reason we like to provide a visualization of them, with full detail in the Github link. In order to provide complementary visualization of the data we have created new figures (shown below) following the reviewers suggestions (figures 6a and b, page 27). These new figures show a subset of the overall network, focusing on the top paths we identified from the Boolean simulations. We will follow the editor guidance for deciding which contents to keep in the main manuscript and which one in the supplementary.

- What do the authors mean by “genomics”? Is this based on RNA-seq data? If so, what tissue was this done on?

Sorry for the confusing terminology. By genomics we were referring only to SNP data. We added a line in figure 1 legend methods that explains genomics refers to single nucleotide polymorphisms (SNPs) typing from DNA arrays.

- What were the ethnic and/or ancestry compositions of the cohorts? The authors do not seem to take into account ancestry as a covariate in their analyses

Yes, such data is collected. In our dataset 99% of the participants were Caucasian, which is expected for MS in Europe, which predominantly affects Caucasians compared to other ethnicities. For this reason, we have not corrected for ethnicity in our study. We have indicated such extend in methods (page 42)

- There seems to be no replication of their findings. The networks identified should be replicated in a second dataset. Alternatively, the authors could use half the dataset to identify networks and the other half of the dataset to validate findings

This is a relevant point. We conducted a partial validation using the single cell analysis from the cytometry. We were interested in conducting a validation in an independent dataset, but no datasets with multiomics are available for MS. Therefore we applied the algorithm to a dataset from the study *Longitudinal multi-omics of host–microbe dynamics in prediabetes* (Zhou et al. 2019). This is explained above and further in the manuscript.

- The authors do not seem to account for treatment as a covariate in their analyses. Furthermore, the DMT given to patients may result in some reverse causation effects (i.e., an individual may have been selected to be on a given DMT based on their clinical outcome), which would greatly confound results.

We agree with the reviewer that disease modifying therapies are important for the analysis. Treatment was included in the clinical variables categorized as low and high efficacy therapy as well as for the response to therapy variables and is described in results. We agree about the reverse causation problem and for this reason we have conducted the analysis based on disease severity, that will stratify DMTs use for low and high efficacy therapy respectively. We have included results for the topological and dynamic analyses of the networks constructed from patients taking low versus high efficacy treatment

Supplementary Figure S2: Comparing the network topology of various subsets of patients from the original datasets: (a,b) disease severity, (c,d) disease subtype, (e,f) efficacy of treatment.

Supplementary Figure S3: Comparison of paths identified for the various subsets of patients from the original datasets. The first page of images shows differences between mild and severe patients, the second page relapse-remitting and progressive MS patients, and the third page patients taking low efficacy and high efficacy treatments. In each comparison, sources from three different layers are shown: (a,b) sources from proteomics, (c,d) sources from genomics, and (e,f) sources from cytomics.
